# Research protocol for an epidemiological study on estimating disease burden of pediatric HIV in Belgaum district, India

**DOI:** 10.1186/s12889-016-3132-8

**Published:** 2016-05-26

**Authors:** Anju Sinha, Anita Nath, Rajeev Sethumadhavan, Shajy Isac, Reynold Washington

**Affiliations:** Indian Council of Medical Research, New Delhi, India; Public Health Foundation of India, Bangalore, Karnataka India; Karnataka Health Promotion Trust, Bangalore, Karnataka India; Karnataka Health Promotion Trust, St John’s Research Institute, Bangalore, Karnataka India

**Keywords:** HIV infection, Pediatric, Burden, Estimation, Case detection

## Abstract

**Background:**

Pediatric HIV is poised to become a major public health problem in India with the rising trend of HIV infection in pregnant women (Department of AIDS Control, Ministry of Health and Family Welfare, http://www.naco.gov.in). There is lack of information on the epidemiology of pediatric HIV infection in India. Existing surveillance systems tend to underestimate the Pediatric burden. The overall aim of the present study is to estimate the disease burden of pediatric HIV among children in Belgaum district in the state of Karnataka in Southern India. An innovative multipronged epidemiological approach to comb the district is proposed.

**Methods:**

The primary objectives of the study would be attained under three strategies. A prospective cohort design for objective (i) to determine the incidence rate of HIV by early case detection in infants and toddlers (0–18 months) born to HIV infected pregnant women; and cross sectional design for objectives (ii) to determine the prevalence of HIV infection in children (0–14 years) of HIV infected parents and (iii) to determine the prevalence of HIV in sick children (0–14 years) presenting with suspected signs and symptoms using age specific criteria for screening. Burden of pediatric HIV will be calculated as a product of cases detected in each strategy multiplied by a net inflation factor for each strategy.

*Study participants* (i) (ii) (iii): HIV infected pregnant women and their live born children (ii) Any HIV-infected man/woman, of age 18–49 years, having a biological child of age 0–14 years (iii) Sick children of age 0–14 years presenting with suspected signs and symptoms and satisfying age-specific criteria for screening. *Setting and conduct:* Belgaum district which is a Category ‘A’ district (with more than 1 % antenatal prevalence in the district over the last 3 years before the study). Age-appropriate testing is used to detect HIV infection.

**Discussion:**

There is a need to strengthen existing pediatric HIV estimation methods in India and other developing countries. We hope that the novel methodology emanating from this study would be applicable for estimating the burden of HIV in other settings and it would be adaptable for estimating the burden of other infectious/chronic diseases. Findings from this study will give future direction to the national program for prevention and control of HIV in India and other developing countries.

**Electronic supplementary material:**

The online version of this article (doi:10.1186/s12889-016-3132-8) contains supplementary material, which is available to authorized users.

## Background

HIV/AIDS is a leading cause of preventable death in children in 42 countries lacking progress towards MDG4 [1]. Advent of HIV threatens advances in child health made over few decades. Globally, there were 2.6 million children living with HIV in 2014, 220,000 new infections among children, and 150,000 AIDS deaths were reported [2].

Global success of prevention of Mother- to- Child transmission has brought down the number of newly infected infants by 58 %, however, only a proportion of the total target women are reached with universal coverage in developing countries. Even under most optimistic scenario for preventive interventions, expected to reach 95 % infected mothers, it is estimated that 1.94 million children will be living with HIV in 2020 [3].

Developing countries account for 95 % of vertical transmission of HIV which has resulted in an increasing number of children with HIV [4]. India has an estimated 220,000 children infected by HIV/AIDS wherein about 55,000 to 60,000 children are born every year to mothers who are HIV infected [5]. The prevalence in India is lower than many countries in the south East Asian region. However, a 1 % increase in the HIV prevalence in adults would result in an additional 5 million infected people [6], because of the large size of the population. There is a rising trend of HIV infection among monogamous pregnant women in the medium and low prevalence states in India; therefore, pediatric HIV is poised to become a major public health problem [7]. This is likely to happen in our society where child bearing is considered essential for a woman and is accorded high priority. This emphasizes the need for better integration of prevention of maternal to child transmission (PMTCT) with maternal and child health services in the country.

From the very beginning of the global response to the HIV/AIDS pandemic, prevention has been marginalized. There is a need to strengthen prevention efforts by using data to determine the most appropriate interventions, address the factors that put women and girls at greater risk and emphasize evidence-based approaches [8]. It is important to estimate the disease burden of Pediatric HIV infection in infants born to known HIV positive mothers, and to start the anti-retroviral treatment (ART) for the infected children at the earliest, so as to save and improve the lives of affected children.

There is lack of information on the epidemiology of pediatric HIV infection in India. Most of the studies that have been done are hospital based being clinical in nature [9]. It is important to diagnose the burden of Pediatric HIV infection in order to develop evidence driven management.

Even though the overall prevalence of HIV in India is 0.27 [10] per cent, the country is divided into three zones based on the prevalence as high, medium and low prevalence states. With migrant populations (travelling from high to low prevalence states) as major drivers of the epidemic, the transmission is expected to be affecting the unsuspecting monogamous women, resulting in a much higher rate of vertical transmission than projected by the sentinel surveillance data. The existing PMTCT services based in the public health system may not detect all the pediatric cases since it does not cover the private health care providers & NGOs etc.

This protocol proposes to use an innovative epidemiologic design with a multipronged approach in the form of three distinct strategies describe under methods that includes active surveillance with involvement of public and private health care facilities, blood banks, involvement of Non Governmental Organizations (NGOs), screening of PLHIVs & children seeking care in specialized clinics) to get an accurate disease burden in Belgaum, Karnataka –a district with HIV prevalence >1 % among pregnant women.

It is hoped that the methodology used in this study could serve as a baseline for planning and implementing HIV prevention and care services in districts with high HIV prevalence in India. It would be applicable for estimating the burden of other diseases and may be used as a benchmark.

## Methods

**Fig. 1:**
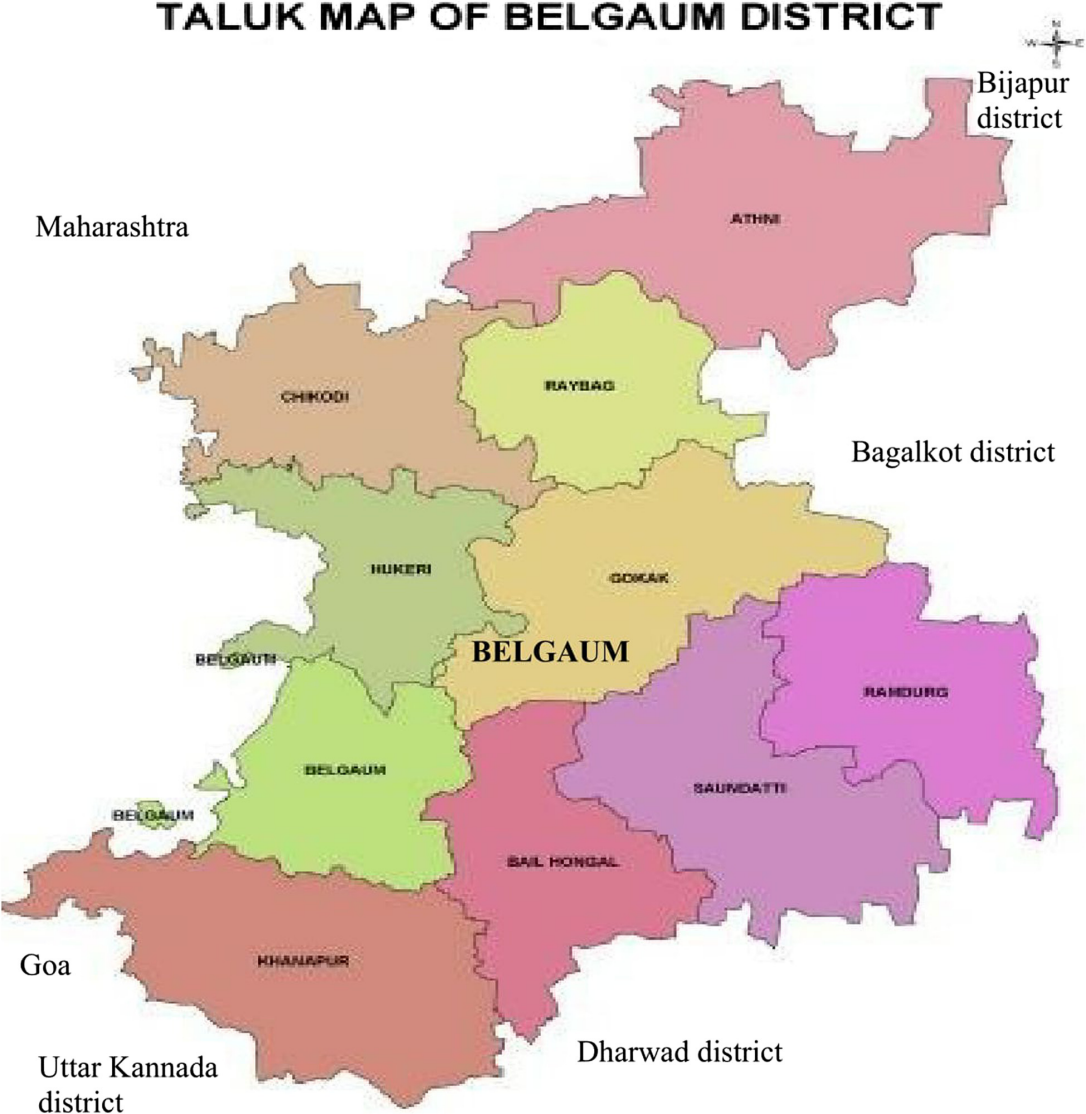
Map of Belgaum District. (Source: www.southindiaonline.com/karnataka/belgaum/locationmap.htm)

**Fig. 2:**
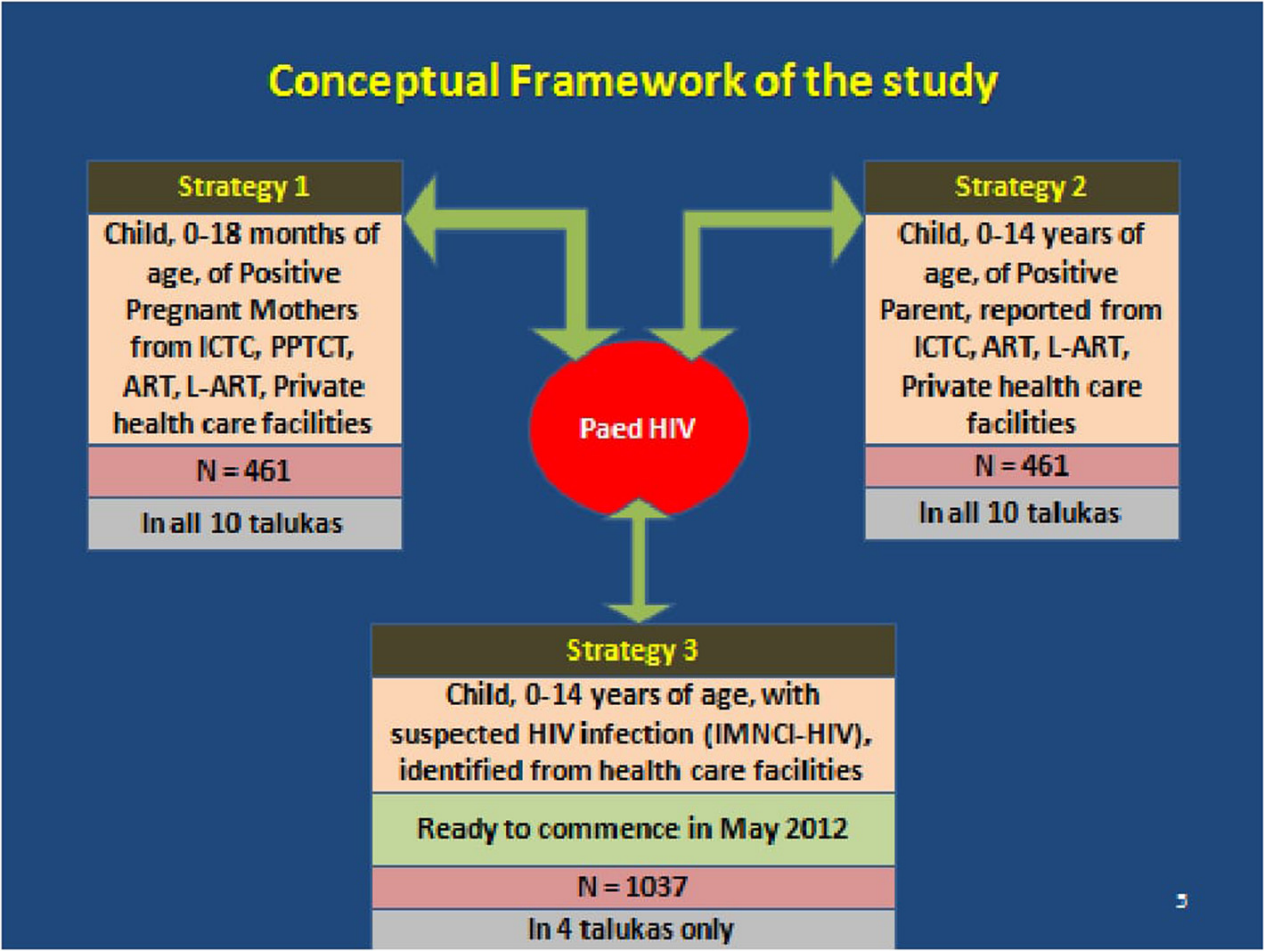
Conceptual framework of the study

The study is implemented in Belgaum district (Fig. 1), which is a Category“A” district as defined by NACO. The district, Belgaum is chosen after conducting a baseline assessment of three ‘A’ category districts in India. The multipronged approach proposed in the study is based on three strategies aimed at different source populations contributing to pediatric disease burden. A conceptual framework of the study is given in Fig. 2.

*Strategy I:* Would use a longitudinal approach aiming early case detection in infants (0–18 months) born to HIV positive women who are registered at PPTCT center in the district. Pregnant women would be followed through their pregnancy & delivery, early testing in infants using DNA PCR dry blood spot (DBS) method would be conducted at 6–8 weeks, & 6 months of age for detection of HIV infection. Infants would be followed up for HIV testing till the age of 20 months.

*Eligibility for strategy I*: 1) HIV infected pregnant women residing in any of the 10 ‘taluka’ of Belgaum district, 2) All live born infants born to these pregnant women 3) Mother/parent who are willing to give consent to undertake the age-specific HIV blood test for their infant.

*Strategy II:* Would use a cross sectional approach for case detection in children by family screening of HIV positive parents (PLHAs) referred from ICTC centers, blood banks and community based NGOs in all ten talukas of Belgaum. If a positive male is detected his wife and children would be tested, if a positive female is detected her husband and children would be tested. Age appropriate testing would be conducted in children (DNA PCR DBS for <18 months, and antibody based HIV testing- ELISA for children aged >18 up to 14 years). This would provide a cross sectional prevalence among 0–14 years age group.

*Eligibility for strategy II*:1) Any HIV infected man/woman, of age 18–49 years, having a biological child of 0–14 years, residing in any ‘taluka’ of Belgaum district, 2) Those willing to give consent to test their spouse and children for HIV during the study period.

Strategy III: Would use screening of sick children visiting health care facilities, in four talukas of Belgaum. IMCI HIV criteria has been adapted by Indian experts to include children >5 upto 14 years into the algorithm. Sick children presenting with common signs symptoms, at shortlisted Govt./Pvt. Health facilities would be assessed and screened. Those screened positive would be subjected to age appropriate HIV testing.

*Eligibility for strategy III:* 1) Children 5–14 years fulfilling adapted IMCI HIV criteria, or having one of the ‘special clues’ in the algorithm, 2) Parents who are willing to provide consent undertake the age-specific test for their child.3) Resident of Belgaum district.

### Setting and design

The study is being conducted in Belgaum, a Category ‘A’ district (with more than 1 % antenatal prevalence in the district over the last 3 years before the start of the study) as defined by the National AIDS Control Organization (NACO). The district is divided into ten sub-districts or ‘talukas’ [Fig. 1]. We are using a multipronged approach by adopting three strategies to enhance the detection of pediatric HIV infection [Fig. 2].

During the preparatory phase a mapping of all existing health care facilities (HCFs) in Belgaum district [11] has been conducted by consolidating various secondary sources of information, key informant survey and snow-ball technique [13, ref published report], Government and private HCFs belonging to all ten talukas of Belgaum District providing HIV testing will be covered under strategy I and strategy II. Activities for strategy III will be conducted in four ‘talukas’; HCFs with “in-patient care facility, “an in-house physician or pediatrician”, and “at least 30 pediatric cases in a month” and all government and subsidiary HCFs, are planned to be included. These four ‘talukas’ have been identified based on the HIV prevalence representing each of the low, medium and high prevalence ‘talukas’.

### Sample size

#### Strategy I

The HIV positive pregnant women receive antiretroviral prophylaxis in the district. Assuming a risk of mother to child transmission as 20 %, a 20 % relative precision at 95 % confidence level, the number of children required is 461 with a design effect of 1.2.

#### Strategy II

Assuming that 20 % of the children of parents tested positive are infected, the sample size required at 95 % confidence level with 20 % relative precision is 461 with a design effect of 1.2.

#### Strategy III

Assuming that 10 % of the sick children meeting the criteria for testing at health facilities in the district could be infected, at 90 % confidence level with 20 % precision, sample size required is 1037 with a design effect of 1.2.

### Study enrolment and data collection

*Strategy I:* Aims early case detection in infants (0–18 months) born to HIV positive women registered at PPTCT center in the district by the DNA PCR dry blood spot (DBS) method at 4–6 weeks and 6–9 months of age. The study team will visit the identified health care facilities twice a week. A crude line list will be made from secondary data collected from various HCFs providing HIV testing facilities to all the positive pregnant women in Belgaum district. The list would be refined by removing duplicates and applying eligibility criteria. All HIV infected pregnant women found to be eligible would be contacted over phone (wherever possible) and subsequently visited at their home (if permitted) or elsewhere at her convenience (if home visits are not permitted). Possible deletions could be due to migration, death or wrong addresses. In case of any change in address, this would be updated in the records. The list of eligible contacted women would be updated for any corrections in addresses, landmarks etc. Possible deletions could be due to those testing positive after delivery, MTP/abortion, or wrong reporting of pregnancy. IRB approval is obtained from St. John’s Research Institute, Bangalore. Informed written consent would be obtained from HIV infected pregnant women or their spouses, for enrolling them as a study participant as per ethics guidelines.

Each recruited pregnant women will be visited once in a month until delivery and thereafter till (i) the infant attains 18 months of age, or (ii) till end study, or (iii) the family migrates permanently out of the district, or (iv) the death of the infant. Each mother-infant pair will be given a unique identification number. Demographic information at the household level and Information on mother and infant would be collected.

*Strategy II* Aims for case detection in children born to HIV positive parents referred from ICTC centers, blood banks and NGOs in the districts by age appropriate testing (DNA PCR DBS for <18 months, antibody based HIV testing- ELISA/Rapid Tests for children aged >18 months up to 18 years).

Public and private Health care facilities identified through the mapping exercise, that provide HIV testing will be visited twice a week, by the study team, to collect information about HIV infected individuals aged 18–49 years. The list would be refined after removing duplicates etc as described under strategy I. These individuals would be contacted to find out those having a biological child aged 0–14 years (wherever known). The spouse and all children in the nuclear family of the positive person (Male or Female) would undergo the age-appropriate HIV test. Data will be collected on a household form; during the first contact, or as and when the family members complete testing. A unique identification number for the positive person as well as other family members testing positive would be created.

*Strategy III* Aims for case detection in sick children (0 months to 18 years) presenting with suspected signs symptoms at selected heath care facilities, & screened positive by the modified IMNCI HIV algorithm. Age appropriate HIV testing would be done.

Generic WHO/UNICEF IMCI-HIV algorithm has been modified for the ICMR study by an expert committee to include children up to the age of 14 years. Sign symptoms from the IAMI and ‘special clues’ (elements from case history) have been added to make the screening for HIV age appropriate. Operational definitions have been developed for each criteria included in the screening algorithm. All participating physicians from the selected health care facilities have been trained in the use of the screening algorithm. These facilities and doctors would be visited once a week by the research team. Information about children satisfying the screening criteria for HIV testing and their referrals, would be collected, children would be tested and tracked for test results.

### Data management & statistical analysis

Data Management: Data entry will be done using Microsoft Access Data will then be exported to SPSS software version 22.0. for further analysis.

Data Analysis: Will be done as per the Statistical Analytical Plan developed by the research team. Each ‘taluka’ is regarded as a cluster for analysis purposes. Crude and adjusted odds ratios will be computed to assess presence of association of child positivity with different variables and the magnitude of statistical significance. A P-value of less than 0.05 at 95 % CI data will be considered as statistically significant.

### Primary outcomes

*Strategy* I: Cumulative incidence will be calculated as the number of new infections per total number of children at risk. Age-specific cumulative incidence rate of HIV infection will also be calculated. The denominator for all the indicators is total person-months. A child will be at risk till first positive result by any test at any age, by age appropriate testing. For censored observations, time will be duration of follow up.

*Strategy II*: Prevalence of HIV infection among children 0–14 years of age would be calculated. Two levels of analysis are proposed using the following denominators: (i) Total children (0–14 year) tested, and (ii) Total children (0–14 year) enrolled (as refusals for testing are expected).

*Strategy III*: Prevalence of HIV among sick children 0–14 years of age would be calculated. Analysis would be conducted at two levels; using the denominators of total number of children 0–14 years who have been enrolled or tested.

*From all the strategies*: Burden of pediatric HIV in the population can be calculated as a product of cases detected in each strategy multiplied by the Net inflation factor, and then consolidated for the total study.

### Determination of ‘Net inflation factor’

Strategy I: The net inflation factor would be derived based on the estimated number of pregnancies in the district, and proportion of un-tested pregnancies, pregnant mothers not enrolled in the study and un-tested children.

Strategy II: based on the actual/projected 18–49 year population during the study period, estimated number of 18–49 year population having a child of age 0–14 years, proportion of eligible index persons not enrolled in the study and proportion of eligible children not tested.

Strategy III : based on actual/projected 0–14 year population during the study period, estimated number of 0–14 year children experiencing any morbidity, estimated morbid children reaching a HCF for care, estimated children satisfying screening algorithm, and suitable inflation factors for geographical and institutional factors, morbid children not reaching selected HCF but reaching other facilities in the district, un-screened, non-enrolled and untested children. (Further details on calculation of Net inflation factor and burden of pediatric HIV are given as Additional file 1).

### Secondary outcomes

The following indicators will be analyzed and inferred upon: Child mortality rates- Infant mortality rate, Neonatal mortality rate, Early neonatal mortality rate & Perinatal mortality rate. Pregnancy wastage: Abortion and stillbirth rate; maternal mortality rate and its causes.

The results are also expected to inform the reasons for gap or delay between detection of HIV, pre-anti-retroviral treatment registration and anti-retroviral treatment initiation. It is also envisaged to retain and follow up this cohort of positive women and their children for program relevant studies in the next phase.

## Discussion

The present study seeks to measure the burden of pediatric HIV in a district of high HIV prevalence. Prevalence reported by ongoing surveillance underestimates the burden. Effort is being made in the current study to include health care facilities belonging to the private health system, and also the NGOs & CBOs. Three strategies have been designed to cover all the age groups which fall in the pediatric range; as well as to include all new and pre-existing HIV infections. It is hoped that the novel methodology emanating from this study would be applicable for estimating the burden of HIV in other settings and it would be adaptable for estimating the burden of other infectious/chronic diseases. Knowledge that is generated in this study could be used to strengthen existing pediatric HIV intervention strategies and design novel measures towards prevention and control of HIV infection in India and other developing countries.

## Current status

The study is nearing completion. The development of study database and data cleaning is currently going on.

## Abbreviations

ART, antiretroviral therapy; DAPKU, District AIDS prevention and control unit; DBS, dot blood spot; DHO, District Health Officer; DNA PCR, deoxyribonucleic acid polymerase chain reaction; ELISA, enzyme linked immune sorbant assay; HCF, health care facility; ICMR, Indian Council of Medical Research; ICTC, integrated counseling and testing centre; IMCI, integrated management of childhood illnesses; IRB, Institutional Review Board; KSAPS, Karnataka State AIDS Prevention Society; MDG, millennium development goals; MTP, medical termination of pregnancy; NACO, National AIDS Control Organization; NGO, Non-Governmental Organization; PLHIV, people living with HIV; PMTCT, prevention of mother to child transmission; PPTCT, prevention of parent to child transmission; UNICEF, United Nations Children Fund; WHO, World Health Organization

## Additional file

Additional file 1: Calculation of Net Inflation factor. (DOC 175 kb)
