# Supplementary material for: Research protocol for an epidemiological study on estimating disease burden of pediatric HIV in Belgaum district, India
Source: BMC Public Health. 2016 May 26;16:446. doi: 10.1186/s12889-016-3132-8 (PMC4882840; doi:10.1186/s12889-016-3132-8)
Supplement: Additional file 1: — Calculation of Net Inflation factor. (DOC 175 kb) [file 12889_2016_3132_MOESM1_ESM.doc]

**Additional file 1**

**Appendix 1: Calculation of Inflation factor**

***A. From strategy 1.***

For conceptualizing this analysis, consider the below flow diagram.

Estimated No. of pregnant women in the district per year (A)

No. of pregnant women tested (B)

No. of pregnant women not tested (A-B)

No. of HIV positive pregnant women among tested (C)

No. of HIV positive pregnant women among not tested (C/B)*(A-B)

No. of HIV positive pregnant women enrolled (D)

No. of HIV positive pregnant women whose child was tested (E)

Total No. of HIV positive pregnant women in the district C + (C/B)*(A-B)

No. of HIV negative pregnant women (B-C)

No. of HIV positive women not enrolled (C-D)

No. of HIV positive women whose child was not tested (D-E)

Figure 4.Flow diagram for analysis of Strategy 1 for secondary objective 1.

From the above flow diagram:

1. Estimated No. of pregnant women in the district per year = A.
2. No. of pregnant women tested = B.
3. No. of pregnant women not tested = A-B.
4. No. of HIV positive pregnant women among tested = C.
5. HIV positivity among pregnant women = C/B.
6. Expecting same proportion of HIV positivity, No. of HIV positive pregnant women among the non-tested = (C/B)*(A-B).
7. Total No. of HIV positive pregnant women in the district = C + (C/B)*(A-B)
8. No. of HIV positive pregnant women enrolled in the study = D.
9. No. of enrolled HIV positive pregnant women whose child was tested = E.
10. Inflation factor = (8)/(9)*(7)/(8) = (D/E)*[{C + (C/B)*(A-B)}/D] = (A*C)/(B*E).

***B. From strategy 2.***

For conceptualizing this analysis, it is important to consider the below flow diagram.

18-49 year population in the district (A)

No. of 18-49 year HIV positive adults having a child of age 0-14 years enrolled (D)

No. of 18-49 year HIV positive adults having a child of age 0-14 years not enrolled (C-D)

No. of children 0-14 years among enrolled adults (E)

No. of children 0-14 years among adults not enrolled

(E/D)*(C-D)

No. of children 0-14 years tested (F)

No. of children 0-14 years positive (G)

Total No. of children 0-14 years for 18-49 year HIV positive adults in the district

E + (E/D)*(C-D)

No. of children 0-14 years not tested (E-F)

No. of children 0-14 years negative (F-G)

Estimated HIV positive adults in 18-49 year age group (B)

Estimated 18-49 year HIV positive adults having a child of age 0-14 years (C)

No. of children 0-14 years positive among not tested

(G/F)*(E-F)

Total No. of HIV positive 0-14 years children for enrolled 18-49 year HIV positive adults

G + (G/F)*(E-F)

Figure 5.Flow diagram for analysis of Strategy 2 for secondary objective 1.

From the above flow diagram:

1. Total 18-49 year adult population in the district = A.
2. Estimated HIV positive adults in 18-49 year age group = B.
3. Estimated 18-49 year HIV positive adult having a child of age 0-14 years = C.
4. No. of 18-49 year HIV positive adult having a child of age 0-14 years enrolled = D.
5. No. of 18-49 year HIV positive adult having a child of age 0-14 years not enrolled = C-D.
6. No. of children 0-14 years of age for enrolled adults = E.
7. Average no. of children per enrolled adult = E/D.
8. Expecting same proportion of children for adults not enrolled, No. of children 0-14 years of age for adults not enrolled = (E/D)*(C-D).
9. Total No. of children 0-14 years of age for 18-49 year HIV positive adult in the district = E + (E/D)*(C-D).
10. Inflation factor for adults not enrolled =
11. No. of children 0-14 years of age for enrolled adults tested = F.
12. No. of children 0-14 years of age for enrolled adults not tested = E-F.
13. No. of children 0-14 years of age for enrolled adults tested positive = G.
14. HIV positivity among the tested children = G/F.
15. Expecting same proportion of HIV positivity among children not tested, No. of HIV positive 0-14 years children among untested children = (G/F)*(E-F).
16. Total No. of 0-14 years HIV positive children for enrolled 18-49 year HIV positive adult = G + (G/F)*(E-F).
17. Inflation factor for children not tested =
18. Net inflation factor =

***C. From strategy 3.***

For conceptualizing this analysis, it is important to consider the below flow diagram.

0-14 year population in the district (A)

Estimated no. of 0-14 year sick children*, reaching any Health Care Facility in the district (D)

No. of sick children* 0-14 years, reached an identified Health Care Facility (E)

Estimated no. of 0-14 year children experiencing any morbidity in the district (B)

Estimated no. of 0-14 year sick children* in the district (C)

Geographical inflation factor (for taluka selection)

Institution inflation factor (for Health Care Facility selection)

No. of sick children* 0-14 years, not reached an identified Health Care Facility (D-E)

No. of sick children* 0-14 years, screened at identified Health Care Facility (F)

No. of sick children* 0-14 years, enrolled and tested (H)

No. of sick children* 0-14 years, found eligible and enrolled (G)

Inflation factor for the unreached children

No. of sick children* 0-14 years, not screened at identified Health Care Facility (E-F)

Inflation factor for the unscreened children

No. of sick children* 0-14 years, eligible-but-not-enrolled (F-G)

Inflation factor for the non-enrolled children

No. of sick children* 0-14 years, eligible-but-not-enrolled (G-H)

Inflation factor for the untested children

Figure 6.Flow diagram for analysis of Strategy 3 for secondary objective 1.

As in Strategy 1 and 2, the following inflation factors need to be considered:

1. Geographical Inflation factor for taluka selection =
2. Institutional inflation factor for Health Care Facility selection =
3. Inflation factor for the unreached children =
4. Inflation factor for the unscreened children =
5. Inflation factor for the non-enrolled children =
6. Inflation factor for the untested children =
7. Net Inflation factor =

**Burden of pediatric HIV in the district = Cases detected in each strategy x (Net) Inflation factor.**

***Source of data:***

- Estimates from secondary sources like projected Census data, DLHS, IMCI Morbidity profile, General Population Survey (GPS), etc.
- Actual study data.

Fig 1.
